# AMPK activation overcomes anti-EGFR antibody resistance induced by *KRAS* mutation in colorectal cancer

**DOI:** 10.1186/s12964-020-00584-z

**Published:** 2020-07-23

**Authors:** Hua Ye, Yi Liu, Kefeng Wu, Hui Luo, Liao Cui

**Affiliations:** 1 Guangdong Key Laboratory for Research and Development of Natural Drugs, Guangdong Medical University, Zhanjiang, 524023 Guangdong Province China; 2 Institute of Marine Biomedical Research, Guangdong Medical University, No.2 Wenming East Road, Zhanjiang, 524023 Guangdong Province China; 3 Southern Marine Science and Engineering Guangdong Laboratory (Zhanjiang), Zhanjiang, 524023 Guangdong Province China

**Keywords:** CRC, EGFR, AMPK

## Abstract

**Background:**

Colorectal cancer (CRC) is associated with resistance to anti-epidermal growth factor receptor (EGFR) antibodies (both acquired and intrinsic), owing to the amplification or mutation of the *KRAS* oncogene. However, the mechanism underlying this resistance is incompletely understood.

**Methods:**

DLD1 cells with WT (+/−) or *KRAS* G13D mutant allele were treated with different concentrations of Cetuximab (Cet) or panitumumab (Pab) to study the mechanism underlying the *KRAS* mutation-induced resistance to anti-EGFR antibodies. The function of AMPK in *KRAS* mutation-induced resistance to anti-EGFR antibodies in CRC cells, and the regulatory role of Bcl-2 family proteins in DLD1 cells with WT or mutated *KRAS* upon AMPK activation were investigated. In addition, xenograft tumor models with the nude mouse using DLD1 cells with WT or mutated *KRAS* were established to examine the effects of AMPK activation on *KRAS* mutation-mediated anti-EGFR antibody resistance.

**Results:**

Higher levels of AMPK activity in CRC cells with wild-type *KRAS* treated with anti-EGFR antibody resulted in apoptosis induction. In contrast, CRC cells with mutated *KRAS* showed lower AMP-activated protein kinase (AMPK) activity and decreased sensitivity to the inhibitory effect of anti-EGFR antibody. CRC cells with mutated *KRAS* showed high levels of glycolysis and produced an excessive amount of ATP, which suppressed AMPK activation. The knockdown of AMPK expression in CRC cells with WT *KRAS* produced similar effects to those observed in cells with mutated *KRAS* and decreased their sensitivity to cetuximab. On the contrary, the activation of AMPK by metformin (Met) or 5-aminoimidazole-4-carboxamide ribonucleotide (AICAR) could overcome the *KRAS*-induced resistance to the anti-EGFR antibody in vivo and in vitro. The activation of AMPK resulted in the inhibition of myeloid cell leukemia 1 (Mcl-1) translation through the suppression of the mammalian target of rapamycin (mTOR) pathway.

**Conclusion:**

The results established herein indicate that targeting AMPK is a potentially promising and effective CRC treatment strategy.

Video abstract

## Background

Colorectal cancer (CRC) is the fourth most common malignancy and the second most frequent cause of cancer-related deaths in the United States [1]. The present treatment options for CRC include targeted therapy using monoclonal antibodies against vascular endothelial growth factor (VEGF)-A or epidermal growth factor receptor (EGFR) [2]. However, genetic and epigenetic alterations such as microsatellite instability, mutations in *KRAS*, *BRAF*, and *PIK3CA* genes lead to drug resistance in CRC [3]. *KRAS* mutations result in the overexpression of phosphatidylinositol-4,5-bisphosphate 3-kinase (PI3K)/protein kinase B (AKT) and RAF/mitogen-activated protein kinase (MEK)/extracellular signal-regulated protein kinase (ERK) signaling [4] and impart resistance to anti-EGFR antibody therapy [5]. However, the exact mechanisms underlying mutant *KRAS*-mediated resistance to anti-EGFR therapy remain unclear. A variety of approaches have been explored to target the mutant *KRAS* gene, including direct inhibition of gene expression [6] and targeting of effector pathways downstream of *KRAS* [7]. Despite these efforts, the *KRAS* mutation is a consistent challenge in the field of oncology, highlighting the need for the discovery of novel mechanistic insights and targeting approaches to resolve *KRAS*-mediated resistance.

Transcriptome and metabolomic analyses have indicated the vital role of *KRAS* mutations in the control of tumor metabolism through the stimulation of glucose uptake [8]. Alteration in energy metabolism, including increased aerobic glycolysis, is a fundamental phenotype of malignant tumors and associated with tumor progression, metastasis, relapse, and chemoresistance [9–11]. AMP-activated protein kinase (AMPK) is a heterotrimeric serine/threonine-protein kinase (STK) that is phosphorylated by its upstream kinase STK11 (LKB1) in response to an increase in cellular AMP/ATP ratio [12]. Activation of AMPK is cytotoxic to various cancer cells and may inhibit tumor growth [13, 14], supporting the role of AMPK as a tumor suppressor and its potential application in cancer therapy and chemoprevention. The activators of AMPK, metformin (Met) and phenformin [15], were shown to reduce tumor growth in the xenograft, transgenic, and carcinogen-induced mouse models of cancer [13, 16]. The extensive research on the safety and use of Met has encouraged the use of this molecule as an anticancer agent [17]. Thus, a better understanding of the mechanism and consequence of AMPK activation in human cancer is important.

Here, we demonstrate that *KRAS* mutation in CRC suppressed the activation of AMPK to stimulate the translation of myeloid cell leukemia 1 (Mcl-1) via the activation of the mammalian target of rapamycin (mTOR) pathway. AMPK activation may overcome the *KRAS*-mediated resistance to anti-EGFR antibodies and achieve better therapeutic effects in vitro and in vivo. The results established herein indicate that targeting AMPK is a potentially promising, safe, and effective CRC treatment strategy.

## Methods and materials

### Cell culture and reagents

LIM1215, RKO, HT29, and Difi CRC lines (WT *KRAS*) were a kind gift from the Ludwig Institute (Melbourne, Australia). HCT-116, LoVo, T84 (all *KRAS* G13D mutants), and SW480 (*KRAS* G12V mutant) were purchased from the American Type Culture Collection (ATCC, Manassas, VA, USA). Isogenic DLD1 cells with different genotypes of *KRAS* were commercially available from Horizon Discovery. *Mycoplasma detection tests were performed for CRC cells every 6 months*. McCoy’s 5A modified medium was provided by Invitrogen (Carlsbad, CA, USA) and used for cell cultivation at 37 °C in a 5% CO_2_ non-humidified incubator. A solution of 1% penicillin-streptomycin prepared by mixing penicillin (100 units/mL) and streptomycin (100 μg/mL; Invitrogen) and 10% fetal bovine serum (FBS; HyClone, Logan, UT, USA) were supplemented in the medium.

The reagents used in the study included cetuximab (Cet, Merk, Kenilworth, NJ, USA), panitumumab (Pab, Amgen, Thousand Oaks, CA, USA), 5-Aminoimidazole-4-carboxamide 1-β-D-ribofuranoside, Acadesine, N1-(β-D-Ribofuranosyl)-5-aminoimidazole-4-carboxamide (AICAR), Met, glucose, 3-Bromopyruvate (3-BrPA) (Sigma-Aldrich, St Louis, MO, USA).

### Synthesis and transfection of shRNAs, lentivirus, and retrovirus

The shRNA plasmids of lentiviruses were provided by Open Biosystems (Thermofisher, Shanghai, China). As per the manufacturer’s protocol, effective transfection reagent (Qiagen) was used to produce lentiviral particles by co-transfecting 293 T cells with pMD2.G and pSPAX2 (packaging plasmids of lentiviruses) and shRNA plasmids. Retrovirus particle production was conducted as previously described using pBABE-puro (#1764), pBABE-puro/Kras WT (#46745), and pBABE-puro/KRASG12V (#46746) (Addgene, Cambridge, MA, USA) [18].

### Plasmids, siRNA transfection

The plasmids and siRNA transfection were conducted by using lipfectamin 2000 (ThermoFisher, Waltham, MA, USA) as described by the manufacturer. The pcDNA3.1 and pcDNA3.1 Mcl-1 (#25375) were purchased from Addgene. The siRNAs for TSC2 and PUMA were purchased from Sigma-Aldrich.

### MTS and ATP assay

For cell survival analysis, 1 × 10^4^ cells/well were seeded in 96-well plates. At different time points, 3-(4,5-dimethylthiazol-2-yl)-5-(3-carboxymethoxyphenyl)-2-(4-sulfophenyl)-2H-tetrazolium (MTS) assay was performed using the MTS assay kit (Thermofisher) according to the manufacturer’s instructions. The ATP luminescence assay was performed using CellTiter-Glo assay (Promega, Madison, WI, USA) as described by the manufacturer. Chemiluminescence and luminescence were measured by using Wallac Victor 1420 Multilabel Counter (Perkin Elmer, Waltham, MA, USA). The luminescence units were normalized to the total cell number. Each assay was conducted in triplicate and repeated three times.

#### RNA isolation and reverse-transcription quantitative polymerase chain reaction (RT-qPCR)

TRIzol (Invitrogen) was used for the extraction of total RNA, and RNeasy Mini Kit (Qiagen) was used for RNA purification, as indicated in the manufacturer’s protocol. Power SYBR Green Master Mix (Life Technologies) was used to carry out qPCR. ProtoScript First Strand cDNA Synthesis Kit (New England Biolabs) was used for reverse transcription.

#### Annexin V/propidium iodide staining and apoptosis analysis

Apoptotic levels were assayed using Annexin V and propidium iodide (PI) staining with fluorescein isothiocyanate (FITC)-Annexin Apoptosis Detection Kit I (BD Pharmingen) and flow cytometry. Alternatively, the apoptosis was analyzed using Hoechst 33258 (Sigma-Aldrich) nuclear staining, as described previously [19].

#### Immunoblot analyses

The collected tumor samples were used to prepare specimens. TissueLyzer II (Qiagen) was used for the radioimmunoprecipitation assay (RIPA) buffer-mediated disruption of cells to obtain cell lysates, which were then centrifuged. The Pierce bicinchoninic acid (BCA) Protein Assay Kit (Thermo Scientific) or Bradford Protein Assay Kit (Bio-Rad) was employed to determine concentrations of proteins. Immunoblot assay was used as previously described [20] in the presence of antibodies against cleaved caspase-3, Bid, Bim, AMPK, p-AMPK, β-actin (Sigma-Aldrich), Mcl-1 (BD Biosciences), Bax, Bad, Bcl-xL, Bcl-2 (Agilent DAKO, USA), PUMA, and p-S6k, S6k, 4EBP1, p-4EBP1, TSC1, TSC2 (Abcam).

#### Mouse tumorigenesis experiments

For all xenograft tests, we used Nu/Nu mouse models (female; age: 5 to 6 weeks; Charles River, Wilmington, MA, USA). Miniature isolation cages under sterile conditions were used to maintain these mice on-site. Mice had constant access to chow and water. Subcutaneous injection on two flanks of each mouse was performed using 5 × 10^6^ DLD1 cells with WT (+/−) and mutated *KRAS* (G13D/−). Mice were intraperitoneally administered with Met in saline (100 mg/kg; 0.9%) every 2 days after 1 week to allow tumor growth. Cet (0.8 mg) was injected every 3 or 4 days. Some mice received a combination of Cet and Met. The treatment was terminated on day 5 or 15 and the tumors were subjected to immunostaining assay or tumor volume investigation, respectively. Tumor growth was monitored every 2 days using calipers in two experimenters who were not blinded. Tumor volume was calculated using the formula, 0.5 × length × width^2^. Tumors collected after sacrificing these mice were excised. Before embedding in paraffin, formalin (10%) fixing was performed for immunostaining. Every procedure was approved by the Animal Care and Use Committee of Guangdong Medical University.

#### Quantification and data analyses

Statistical significance is determined by Student’s t-test (paired) for bar graphs or One-way ANOVA analysis for growth curves with Graphad Prism (v.5).

## Results

### KRAS mutation in CRC suppresses the phosphorylation of AMPK

**Fig. 1:**
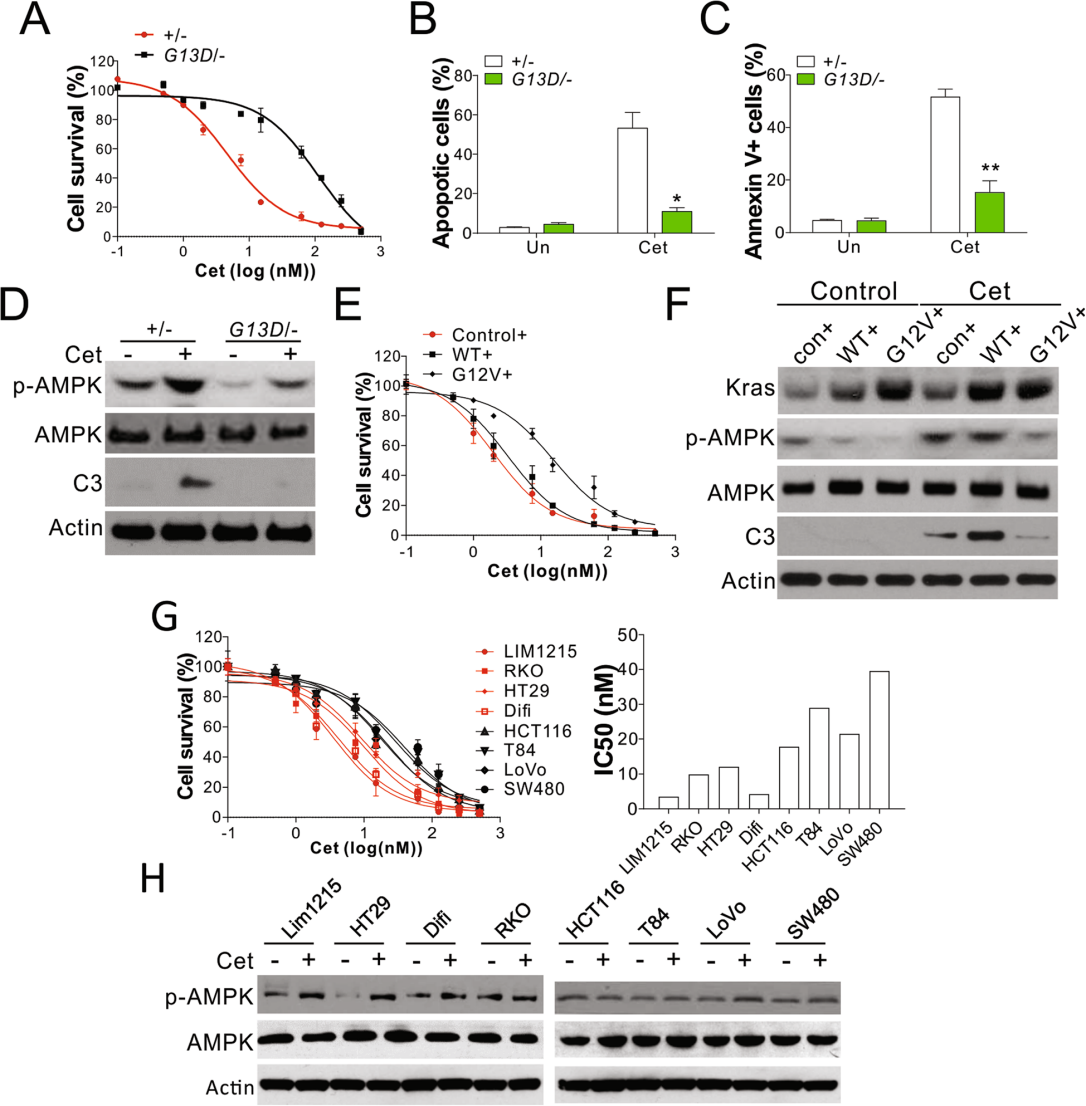
Phosphorylation of AMPK was suppressed by Kras mutation in CRC cells. **a** MTS analysis of DLD1 WT (+/−) and Kras mutation (G13D/−) cells treated with cetuximab (Cet) at the indicated doses for 48 h. **b** Apoptosis in DLD1 WT (+/−) and Kras mutation (G13D/−) cells treated with 5 nM Cet for 48 h was analyzed by nuclear staining with Hoechst 33258. **c** Apoptosis in DLD1 WT (+/−) and Kras mutation (G13D/−) cells treated with 5 nM Cet for 48 h was analyzed by Annexin V staining followed by flow cytometry. **d** Western blot of p-AMPK, AMPK, and Caspase-3 (C3) in the cells treated as in (**b**). **e** DLD1 WT (+/−) cells stably expressing control, Kras WT, or Kras mutant (G12V) by retrovirus transfection were treated with Cet at indicated doses for 48 h. The cell viability was analyzed by MTS assay. **f** DLD1 WT (+/−) cells stably expressing control, Kras WT, or Kras mutant (G12V) by retrovirus transfection were treated with 5 nM Cet for 48 h. The expression of indicated proteins was analyzed western blot. **g** MTs analysis of indicated cells treated with Cet at indicated doses for 48 h. The IC50 was calculated and plotted in the right panel. **h** The expression of p-AMPK, AMPK in the indicated cells treated with 5 nM Cet. Each experiment was repeated for 3 times. *, *p* < 0.05; **, *p* < 0.01

To study the underlying mechanism that the *KRAS* mutation-induced resistance to anti-EGFR antibodies, DLD1 cells with WT (+/−) or *KRAS* G13D mutant allele were treated with different concentrations of Cet or Pab. As a result, we found that Cet and Pab substantially suppressed the growth of DLD1 WT cells in a dose-dependent manner (Fig. 1a, Fig. S1A). In contrast, DLD1 cells with mutated *KRAS* showed resistance to Cet and Pab treatment (Fig. 1a, Fig. S1A). Anti-EGFR antibodies are known to induce cancer cell death via apoptosis [21, 22]; we, therefore, investigated the expression of apoptotic signals in CRC cells treated with Cet or Pab. Treatment with 5 nM Cet or 10 nM Pab markedly induced death in WT DLD1 cells with characteristics of apoptosis (Fig. 1b, Fig. S1B), positive Annexin V staining of plasma membrane (Fig. 1c), and cleavage of caspases-3 (Fig. 1d, Fig. S1C). The apoptotic signals were low in DLD1 cells with mutated *KRAS* after treatment with the same dose of Cet or Pab (Fig. 1b-d, Fig. S1B,C), indicative of the anti-apoptotic effect of this mutation that mediates resistance to anti-EGFR antibodies. We also analyzed the expression and phosphorylation level of AMPK in DLD1 cells with WT or mutated *KRAS*. No significant change in AMPK expression was observed between WT and mutated cells after Cet or Pab treatment (Fig. 1d, Fig. S1C). However, the treatment with anti-EGFR antibodies induced phosphorylation of AMPK in WT cells, and this effect was compromised in DLD1 cells with *KRAS* mutation (Fig. 1d, Fig. S1C). Therefore, *KRAS* mutation suppressed the activation of AMPK. To confirm the effect of *KRAS* mutation on AMPK activation, we transfected DLD1 WT (+/−) cells with WT or mutant *KRAS* (G12V) using retrovirus transfection. The transfection of cells with mutant *KRAS* (G12V) resulted in significant suppression of their sensitivity to Cet and decreased the apoptosis of cells as compared with DLD1 WT cells; this effect was absent in DLD1 cells transfected with WT *KRAS* (Fig. 1e, Fig. S1D,E). Furthermore, we failed to observe AMPK phosphorylation induced by Cet treatment in the cells transfected with *KRAS* mutant gene as compared with those expressing WT *KRAS* (Fig. 1f). We also analyzed the activation of AMPK in eight different CRC lines, including LIM1215, RKO, HT29, and Difi (*KRAS* WT), HCT-116, T84, LoVo (all *KRAS* G13D mutants), and SW480 (*KRAS* G12V mutant). The cell lines with *KRAS* mutations were less sensitive to Cet-induced death and had higher IC50 values (Fig. 1g). Among those *KRAS* WT cells, RKO and HT29 had relatively higher IC50 values, which might due to the BRAF mutation (Fig. 1g). Moreover, Cet-induced phosphorylation of AMPK was lower in the cells with *KRAS* mutations than in other cell lines (Fig. 1h). Taken together, *KRAS* mutations in CRC cells suppressed the activation of AMPK pathway in response to anti-EGFR antibody treatment.

#### Glycolysis is essential for mediating *KRAS* mutation-induced anti-EGFR resistance

**Fig. 2:**
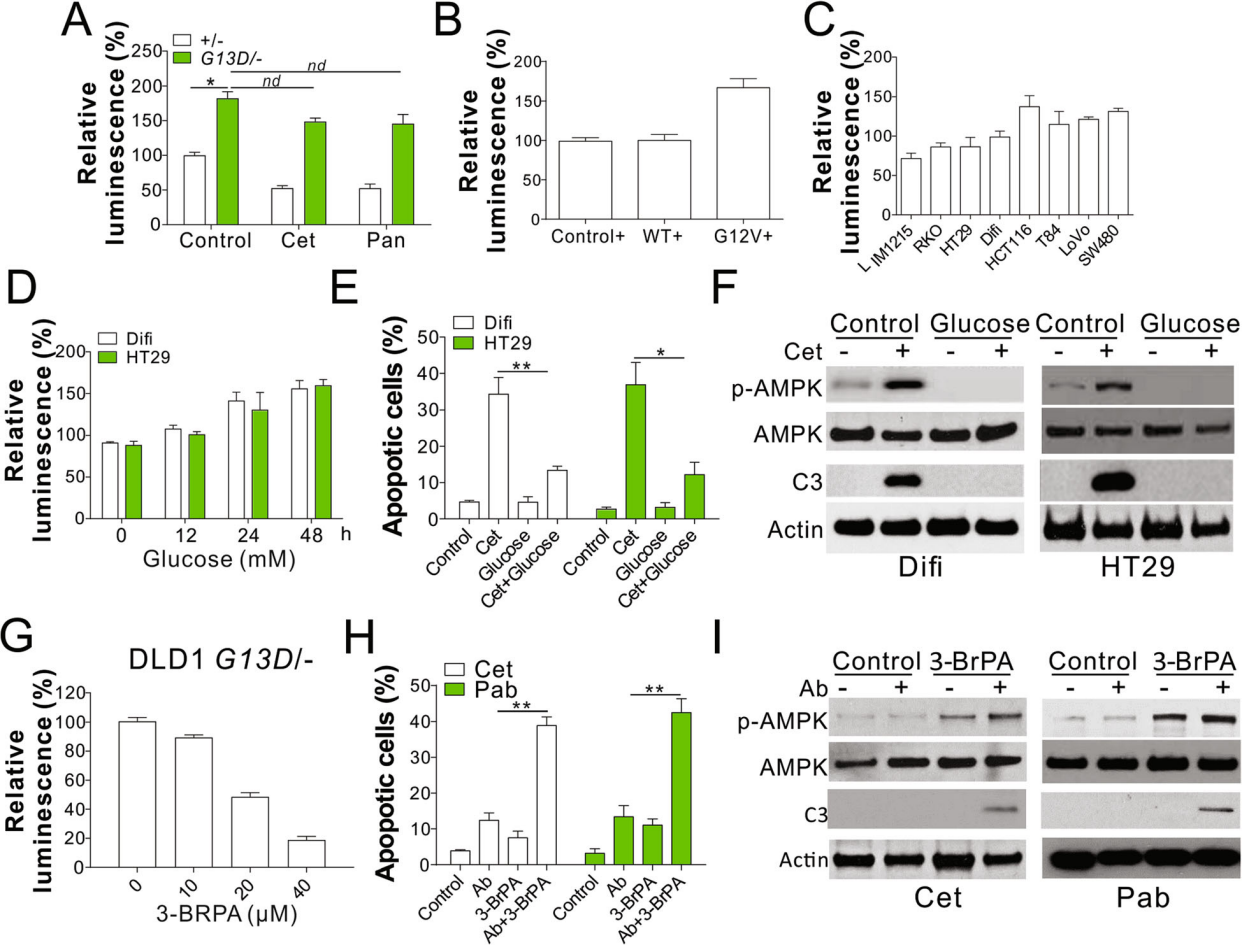
Kras mutation suppressed the AMPK activation by glycolysis. **a** The cellular ATP level of DLD1 WT and Kras mutation cells was analyzed by luminescence assay. **b** The cellular ATP level in DLD1 WT (+/−) cells stably expressing control, Kras WT, or Kras mutant (G12V) by retrovirus transfection (**c**) The cellular ATP level in the indicated cell lines. **d** The cellular ATP level of HT29 and Difi cells cultured in media containing 20 mM glucose at indicated time. **e** HT29 and Difi cells cultured in the media with or without 20 mM glucose were treated with Cet (15 nM for HT29; 5 nM for Difi) for 48 h. The apoptosis was analyzed by Hoechst 33258 staining. **f** Western blot of indicated proteins in HT29 and Difi cells treated as in (**e**). **g** The cellular ATP level of DLD1 Kras mutated treated with 10 μM 3-BrPA for indicated time points. **h** The apoptosis of DLD1 Kras mutated cells treated with 10 μM 3-BrPA in combined with 5 nM Cet or 10 nM panitumumab (Pab). **i** Western blot of indicated proteins in DLD1 Kras mutated cells treated as in (**h**). Each experiment was repeated for 3 times. nd, *p* > 0.05; *, p < 0.05; **, *p* < 0.01

**Fig. 3:**
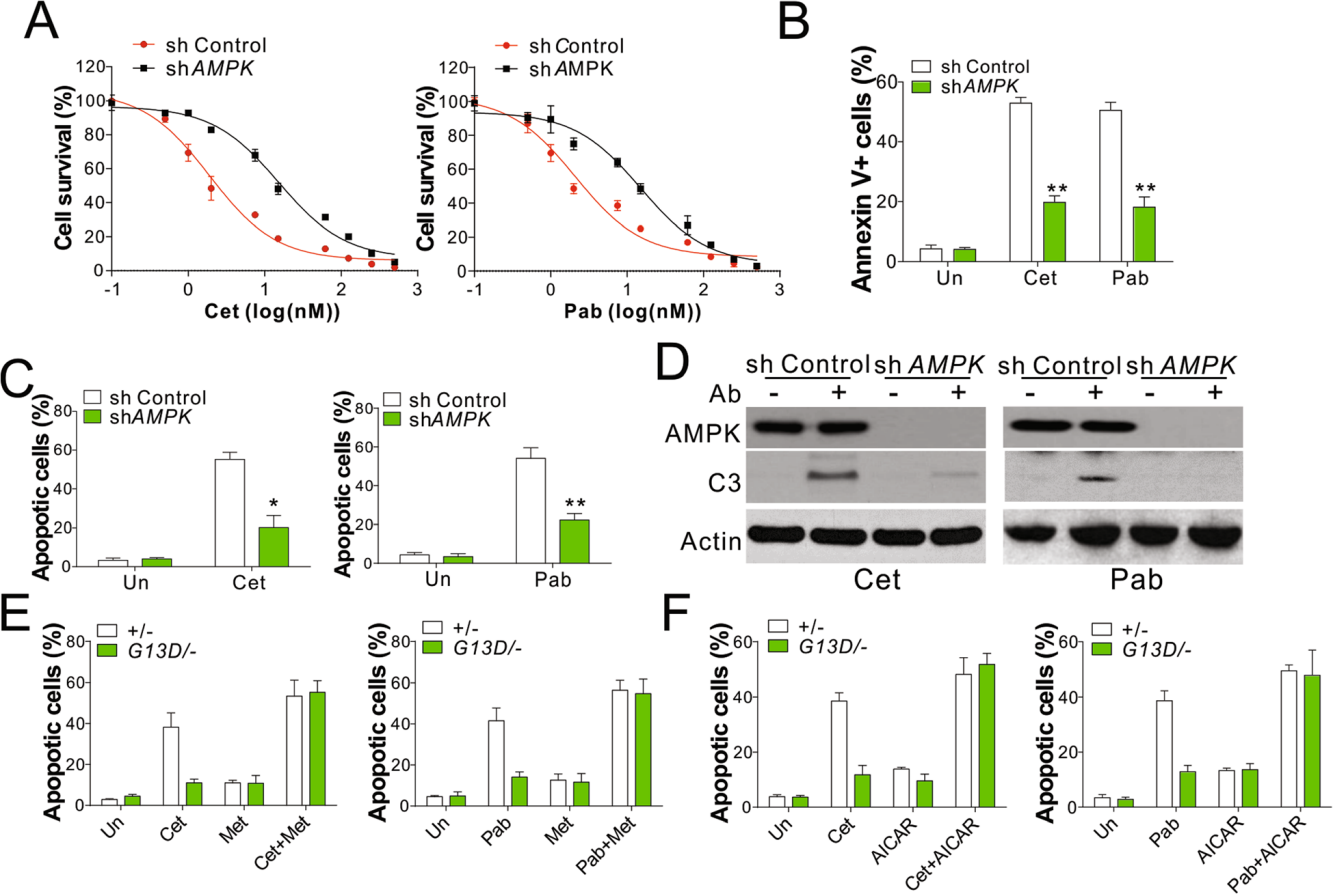
Activation of AMPK reversed the anti-EGFR antibodies resistance induced by Kras mutation. **a** MTS analysis of DLD1 WT (+/−) transfected with control or AMPK shRNA and treated with Cet (left) or Pan (right) at the indicated doses for 48 h. **b** DLD1 WT (+/−) transfected with control or AMPK shRNA were treated with 5 nM Cet or 10 nM Pan for 48 h. The apoptosis was analyzed by annexin-V staining followed by flow cytometry analysis. **c** Hochst 33,258 staining of DLD1 WT (+/−) cells treated as in (**b**). **d** Western blot of indicated proteins in the cells treated as in (**b**). **e** The apoptosis of DLD1 WT or Kras mutation cells treated with 5 μM metformin in combined with 5 nM Cet (left) or 10 nM Pan (right) for 48 h. **f** The apoptosis of DLD1 WT or Kras mutation cells treated with 1 μM AICAR in combined with 5 nM Cet (left) or 10 nM Pan (right) for 48 h. Each experiment was repeated for 3 times. *, *p* < 0.05; **, *p* < 0.01

Previous studies have revealed the suppression of AMPK pathway by aerobic glycolysis [23], which contributes to the development of CRC with KRAS pathway mutations [24]. We, therefore, investigated whether aerobic glycolysis is important for AMPK suppression by *KRAS* mutation. We examined the level of cellular ATP in DLD1 cells expressing WT or mutant *KRAS* using an ATP-based luminescent assay and found that *KRAS* mutation resulted in a two-fold increase in total cellular ATP level as compared with WT cells (Fig. 2a). Transfection of mutant *KRAS* (G12V) in DLD1 WT cells also increased the cellular ATP level as compared with that observed in control cells or those expressing WT *KRAS* (Fig. 2b). Furthermore, the CRC cell lines with *KRAS* mutations had higher levels of cellular ATP than those expressing WT *KRAS* (Fig. 2c), indicative of the production of high levels of ATP in the cells expressing *KRAS* mutations. Treatment of Cet or Pab reduced the ATP level in WT DLD1 cells, but did not have any effects on the cellular ATP level of KRAS mutant (G13D) DLD1 cells (Fig. 3a), suggesting elevation of glycolysis might be the reason of AMPK activation and anti-EGFR drug resistance. To study the function of glycolysis in AMPK activation, we cultured *KRAS* WT cells, Difi and HT29, in the presence of glucose supplementation and found a substantial increase in the level of cellular ATP in both cell lines in a time-dependent manner (Fig. 2d). The supplement of glucose compromised the Cet induced apoptosis (Fig. 2e) and activation of AMPK (Fig. 2f) in Difi and HT29 cells. To confirm the function of glycolysis in AMPK suppression, we treated DLD1 cells expressing mutant *KRAS* with 3-BrPA, a pyruvate analogue with alkylating properties that deplete cellular ATP levels. Pretreatment with 3-BrPA resulted in a significant suppression in cellular ATP levels in DLD1 cells expressing mutant *KRAS* in a dosage-dependent manner (Fig. 2g). Furthermore, 3-BrPA treatment sensitized the mutant cells to Cet- or Pab-induced apoptosis (Fig. 2h,i) and restored the phosphorylation status of AMPK suppressed by *KRAS* mutation (Fig. 2i). Thus, *KRAS* mutation in CRC cells suppressed the activation of AMPK via glycolysis.

### AMPK is necessary for KRAS mutation-mediated resistance to anti-EGFR antibodies in CRC cells

We investigated the function of AMPK in *KRAS* mutation-induced resistance to anti-EGFR antibodies in CRC cells. The depletion of AMPK expression in DLD1 cells by small-hairpin RNA (shRNA) produced effects similar to those observed with *KRAS* mutation, consistent with the retardation of cell death induced by Cet and Pab (Fig. 3a). The absence of AMPK expression also resulted in the suppression of anti-EGFR antibody-mediated apoptosis of DLD1 cells with WT *KRAS* (Fig. 3b-d). We further evaluated whether the activation of AMPK pathway may overcome the resistance to anti-EGFR antibodies induced by *KRAS* mutation and found that the combination treatment of AMPK activator Met or 5-aminoimidazole-4-carboxamide ribonucleotide (AICAR) and anti-EGFR selectively re-sensitized the mutated DLD1 cells to apoptosis induced by antiEGFR antibodies (Fig. 3e,f). In contrast, the treatment with AMPK activator had less effects on WT cells in the presence of anti-EGFR antibody (Fig. 3e,f). Therefore, these results suggest that the activation of AMPK pathway may overcome the drug resistance induced by *KRAS* mutation in CRC.

### AMPK activation suppresses the expression of mcl-1

**Fig. 4:**
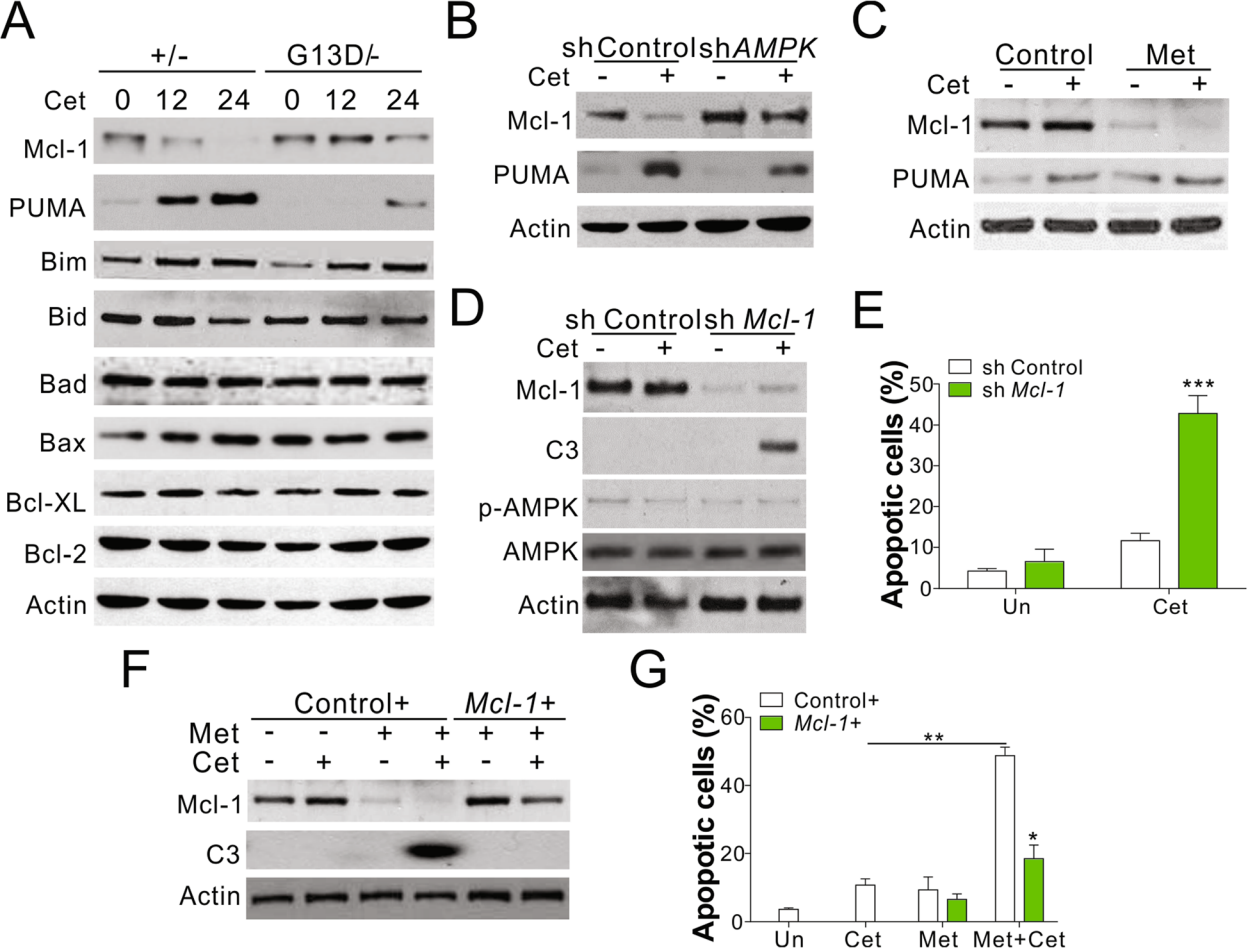
Kras mutation in CRC suppressed AMPK activation to stabilize Mcl-1. **a** Western blot of indicated proteins in DLD1 WT (+/−) and Kras mutation (G13D/−) cells treated with 5 nM Cet for indicated time points. **b** DLD1 WT (+/−) transfected with control or AMPK shRNA were treated with 5 nM Cet for 48 h. The expression of Mcl-1 and PUMA was analyzed. **c** The Mcl-1 and PUMA expression in DLD1 Kras mutation cells treated with 5 μM Met in combined with 5 nM Cet for 48 h. **d** DLD1 Kras mutated cells transfected with control or Mcl-1 shRNA were treated with 5 nM Cet for 48 h. The expression of indicated was analyzed. **e** The apoptosis of DLD1 Kras mutated cells treated as in (**d**). **f** DLD1 Kras mutated cells transfected with control or Mcl-1 plasmids were treated with 5 nM Cet in combined with 5 μM Met for 48 h. The expression of Mcl-1 and caspase-3 (C3) was analyzed. **g** The apoptosis of DLD1 Kras mutated cells treated as in (**f**). Each experiment was repeated for 3 times. *, *p* < 0.05; **, *p* < 0.01; ***, *p* < 0.001

As B cell lymphoma-2 (Bcl-2) family of proteins plays an important role in mediating apoptosis induced by anti-EGFR antibodies and AMPK activator [21, 25], we investigated the regulatory role of Bcl-2 family proteins in DLD1 cells with WT or mutated *KRAS*. Cet treatment induced the expression of PUMA and Bim but suppressed the level of Mcl-1 in WT cells (Fig. 1a). *KRAS* mutation had opposite effects on the induction of PUMA and suppression of Mcl-1 observed after Cet treatment (Fig. 4a). The depletion of PUMA in WT cells resulted in the suppression of Cet-induced apoptosis (Fig. S2A,B), consistent with the previously reported observation about the role of PUMA in anti-EGFR antibody-induced cell death [21]. However, the absence of PUMA had no effect on the apoptosis induced by Cet in combination with Met in DLD1 cells with mutated *KRAS* (Fig. S2C,D). Furthermore, the depletion of AMPK pathway only affected the Cet-induced suppression of Mcl-1 expression but not PUMA induction (Fig. 4b). The activation of AMPK by Met in DLD1 cells with mutated *KRAS* suppressed the expression of Mcl-1 but had no effect on the induction of PUMA expression (Fig. 4c), suggesting that AMPK activation specifically suppressed the expression of Mcl-1 protein. To confirm the role of Mcl-1 protein in KRAS-AMPK-mediated anti-EGFR antibody resistance, we generated a mutated *KRAS* carrying DLD1 cell line with a stable knockdown of Mcl-1 expression. The depletion of Mcl-1 expression resulted in the sensitization of the mutant cells to Cet- and Pab-induced apoptosis (Fig. 4d,e). On the contrary, the increase in the expression of Mcl-1 protein in mutant DLD1 cells compromised the apoptosis induced by the combination of Cet and Met (Fig. 4f,g). Together these results indicate that the abnormal expression of Mcl-1 mediates resistance to antiEGFR antibodies in CRC cells with oncogenic *KRAS*.

### Activation of AMPK suppresses mcl-1 expression by targeting mTOR pathway

**Fig. 5:**
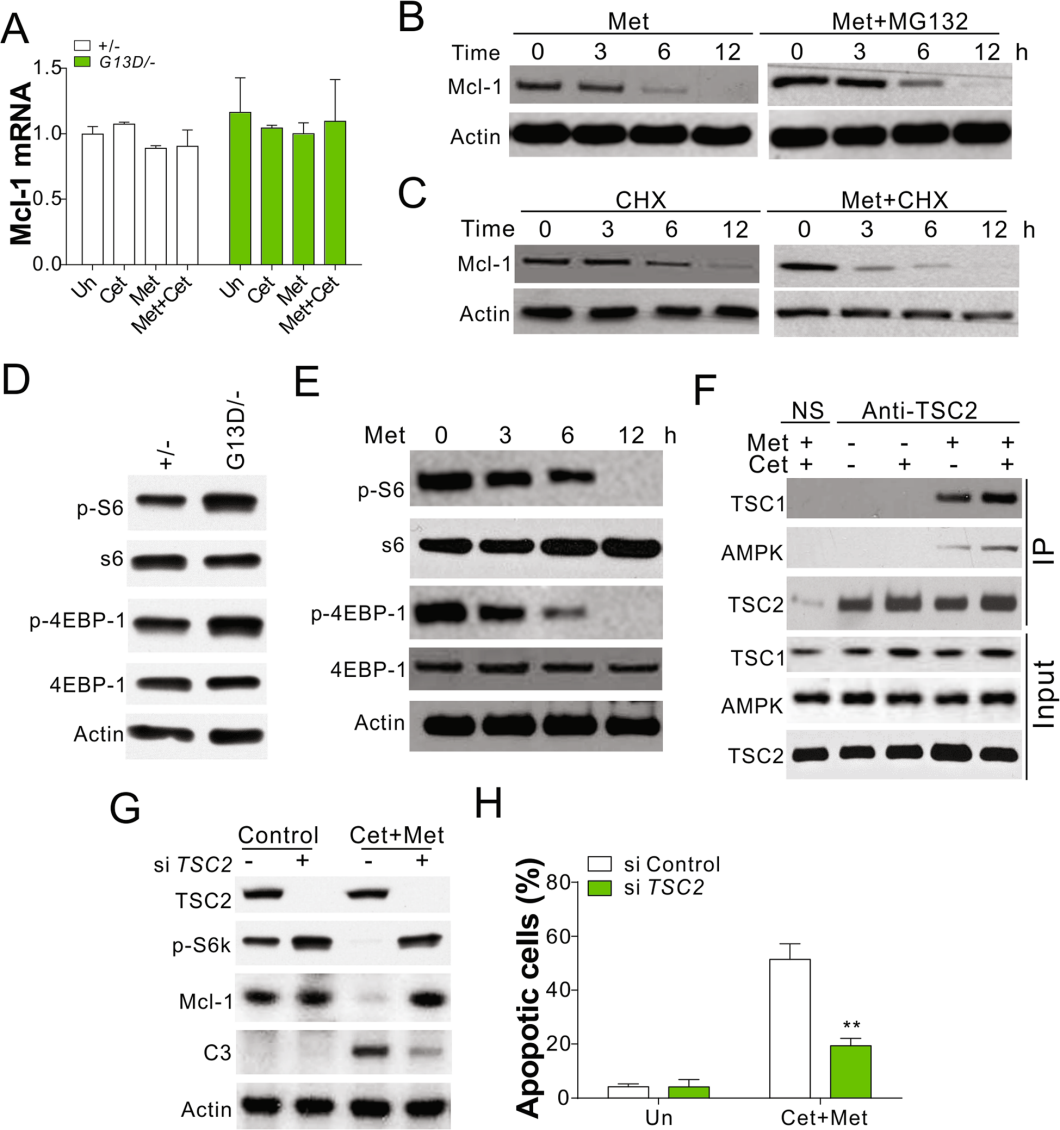
AMPK modulated the translation of Mcl-1 by targeting Mtor pathway. **a** The mRNA level of Mcl-1 in DLD1 WT and Kras mutated cells treated as indicated. **b** The expression of Mcl-1 in DLD1 Kras mutated cells treated with 5 μM Met and/or 5 μM MG132. **c** The expression of Mcl-1 in DLD1 Kras mutated cells treated with 5 μM Met and/or 1 ng/ml CHX. **d** The expression of indicated proteins in DLD1 WT and Kras mutated cells. **e** The expression of indicated proteins in DLD1 Kras mutated cells treated with 5 μM Met for indicated time. **f** The interaction of AMPK, TSC1, TSC2 in DLD1 Kras mutated cells treated with Met and/or Cet was analyzed by immunoprecipitation with anti-TSC2 antibody. **g** The expression of indicated proteins in DLD1 Kras mutated cells transfected with TSC2 siRNA and treated with Met and Cet. (H) The apoptosis in DLD1 Kras mutated cells treated as in (**g**). Each experiment was repeated for 3 times. **, *p* < 0.01

We investigated the mechanism underlying Mcl-1 suppression upon AMPK activation. Treatment with Cet and/or Met had no significant effect on the mRNA level of Mcl-1 in DLD1 cells with WT or mutated *KRAS* (Fig. 5a). Thus, AMPK activation had no effects on Mcl-1 transcription. Pretreatment of DLD1 WT or mutant cells with a protease inhibitor, MG132, had no effect on the Met-mediated regulation of Mcl-1 (Fig. 5b), ruling out the possibility of post-transcriptional modification of Mcl-1. On the other hand, the pretreatment of mutant cells with a translation inhibitor, cycloheximide (CHX), shortened the half-life of the Mcl-1 protein in response to Met treatment (Fig. 5c), indicative of the modulation of *Mcl-1* translation upon *KRAS* mutation. For certain cancers, cap-dependent translation is essential for the efficient translation of *Mcl-1* mRNA [26, 27]. In such situations, the level of Mcl-1 protein reduces after inhibition with mTORC1, resulting in the loss of eukaryotic translation initiation factor 4E-binding protein 1 (4EBP1) expression [27]. Here, we found that the cells with mutated *KRAS* showed a higher level of S6 kinase beta-1 (S6K) and 4EBP1 phosphorylation than WT cells (Fig. 5d). Treatment of DLD1 cells with mutated *KRAS* with Met led to a decrease in S6K and 4EBP1 phosphorylation (Fig. 5e), and this effect correlated with Mcl-1 expression (Figs. 4a and 5b). This observation is suggestive of the suppression of the mTOR pathway in response to the activation of AMPK. The activation of AMPK was shown to result in the formation of a complex with tuberous sclerosis complex 2 (TSC2) that recruits TSC1 and suppresses 4EBP1 and S6K phosphorylation [28]. In the present study, the activation of AMPK by Met in DLD1 cells with mutated *KRAS* resulted in an increase in the interaction between TSC2, TSC1, and AMPK (Fig. 5f). The knockdown of TSC2 expression by small-interfering RNA (siRNA) abolished the apoptotic effects of the combination of Cet and Met in DLD1 cells with mutated *KRAS* (Fig. 5g,h). TSC2 knockdown also recovered the expression of Mcl-1 and phosphorylation of S6K (Fig. 5g). Thus, the activation of AMPK suppressed the mTOR pathway and eventually reduced the translation of Mcl-1 in CRC.

#### In vivo resistance to anti-EGFR antibodies may be overcome via AMPK activation

**Fig. 6:**
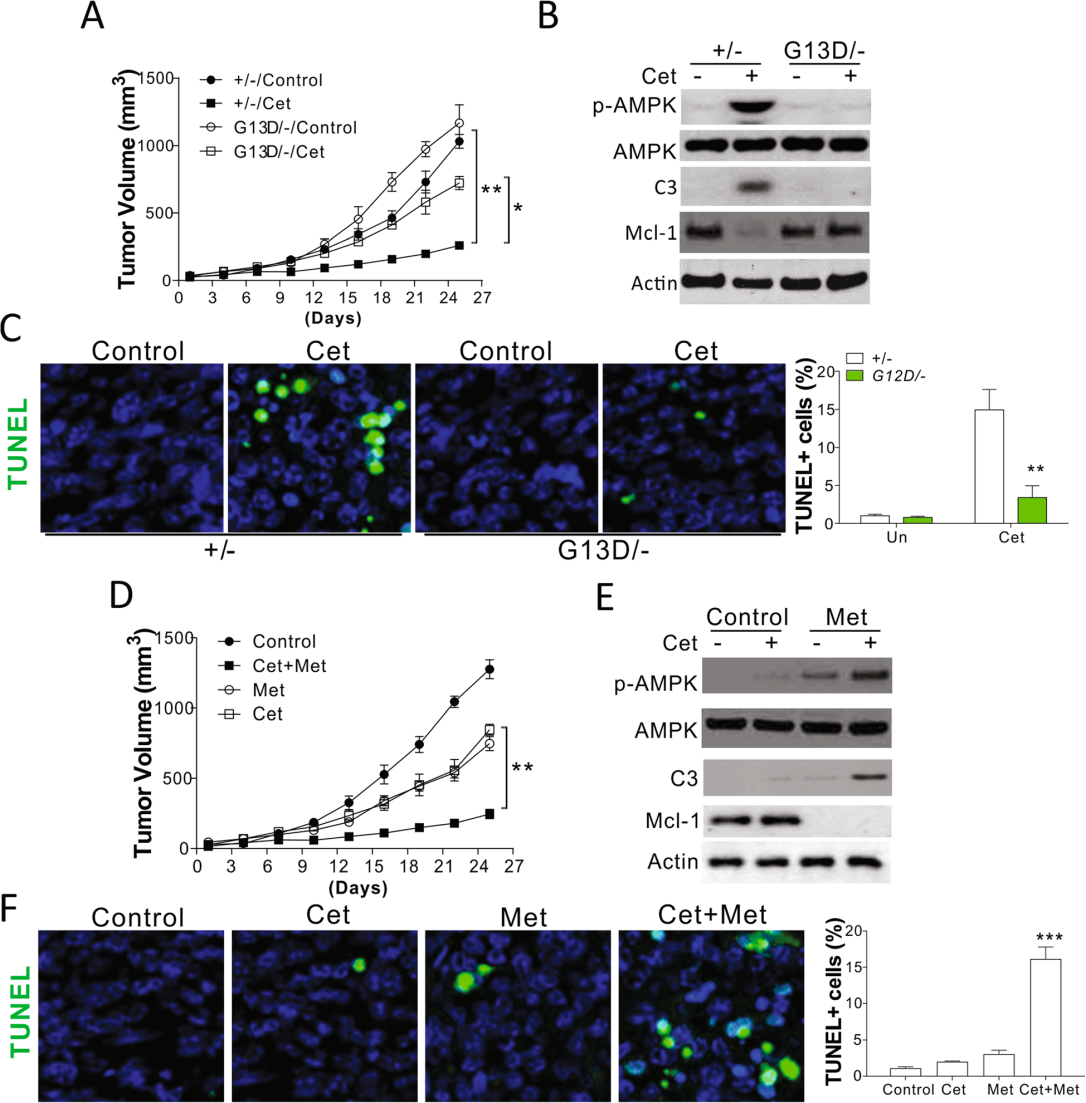
AMPK activation overcome Kras mutation induced drug resistance in vivo. **a** The growth of DLD1 WT and Kras mutated cell implanted xenograft tumor in response to 0.8 mg/kg Cet treatment (*n* = 6). **b** The expression of indicated proteins in representative tumors in the initial 5 days of treatment. **c** The TUNEl staining of tumor sample from (**b**). **d** The growth of DLD1 Kras mutated cell implanted xenograft tumor in response to 0.8 mg/kg Cet and/or 100 mg/kg metformine treatment (*n* = 6). **e** The expression of indicated proteins in representative tumors in the initial 5 days of treatment. **f** The TUNEl staining of tumor sample from (**e**). Panels **b**, **c**, **e**, **f** were repeated for 3 times. *, *p* < 0.05; **, *p* < 0.01, ***, *p* < 0.001

We established a xenograft tumor model with nude mouse using DLD1 cells with WT or mutated *KRAS* to examine the effects of AMPK activation on *KRAS* mutation-mediated anti-EGFR antibody resistance. Consistent with the results of in vitro studies, *KRAS* mutations in tumors induced resistance to Cet treatment; however, no difference in growth was observed for tumors derived from cells with WT or mutated *KRAS* (Fig. 6a). Western blot analysis showed that Cet treatment induced the cleavage of caspase-3, increased AMPK activation, and suppressed Mcl-1 level in tumors obtained from WT cells, and these effects were compromised in tumors derived from the cells with mutated *KRAS* (Fig. 6b). Terminal deoxynucleotidyl transferase dUTP nick end labeling (TUNEL) staining results also showed that the cell death induced by Cet was suppressed in the tumors from cells with mutated *KRAS*, confirming that *KRAS* mutation may induce resistance to anti-EGFR antibody in vivo (Fig. 6c). We evaluated whether the combination of Cet and AMPK activator (Met) may overcome drug resistance in tumors from cells with *KRAS* mutation. As a result, we found that Cet-resistant tumor showed a slight decrease in growth after treatment with Cet or Met but was obviously inhibited in response to the combination treatment (Fig. 6d). On the other hand, the expression of Mcl-1 was inhibited in the resistant tumors by the combination therapy, as evident from analytical results obtained for early collected tumors (Fig. 6e). The cleavage of caspase-3 increased, suggestive of apoptosis activation in response to the combination treatment of Cet and Met (Fig. 6e). This observation was consistent with the results of TUNEL staining (Fig. 6f). Thus, the resistance to anti-EGFR antibodies mediated by *KRAS mutation could be overcome with the use of* AMPK activators to suppress the expression of Mcl-1.

## Discussion

Therapeutic resistance is one of the most significant challenges for targeted therapies [29]. In the present study, we found that *KRAS* mutation in CRC cells imparts resistance to anti-EGFR antibody treatment through the suppression of AMPK activation. *KRAS* mutation in CRC cells increases the level of cellular glycolysis and consequently suppresses the activation of AMPK in response to anti-EGFR antibody treatment. Our results show that the combination treatment with an AMPK activator, Met or phenformin, may re-sensitize the cells with mutated *KRAS* to anti-EGFR antibody-induced apoptosis, suggestive of the potential of this strategy to overcome the drug resistance induced by *KRAS* mutation. The suppression of AMPK expression by *KRAS* mutation may lead to the activation of the mTOR pathway and increase the translation of the Mcl-1 protein.

*KRAS* is the isoform commonly mutated in the pancreas, lung, and colon cancers [30]. Retrospective analyses of clinical data revealed the alterations in *KRAS* oncogene that are mostly responsible for mediating resistance to anti-EGFR antibodies [31]. Previous studies have established that *KRAS* G12D mutation rewired the anabolic glucose metabolic network in multiple cancers that is important for tumor growth [8, 24]. However, whether glycolysis stimulated by *KRAS* mutation contributes to drug resistance is largely unknown. It was reported that the metabolic shift driven by high ATP levels is associated with the progression of acquired chemoresistance of cancer cells [11]. Our data revealed the elevated level of aerobic glycolysis in CRC cells with mutated *KRAS* and demonstrated the sensitization of mutant cells through the inhibition of glycolysis. These observations suggest that the increase in aerobic glycolysis in cancer cells may provide the extra amount of ATP needed for the survival of cells with mutated *KRAS* under stress. The elevated level of cellular ATP in cells with mutated *KRAS* may suppress the activation of AMPK, leading to resistance to anti-EGFR antibodies.

Other than *KRAS* mutation, BRAF, the downstream of *KRAS*, is also frequently mutated in the CRC patients (around 10%) [32]. The mutation of BRAF was also reported to be another vital obstacle for anti-EGFR antibodies-based therapy [32, 33]. In our study, we found that the *BRAF* mutated cells, including HT29 and RKO cells, had relative higher IC50 of cetuximab than the *KRAS*/*BRAF* WT cells, Difi and Lim1215 cells (Fig. 1g), which indicated that BRAF mutation also contributes to the anti-EGFR antibodies resistance in CRC cells. The AMPK activation by Cet was also found in these two BRAF mutated cell lines (Fig. 1h), indicating *BRAF* might be the downstream effector of AMPK. Exactly, it has been reported that AMPK activation leads to phosphorylation of BRAF and impairs its oncogenic effects [34]. Therefore, suppression of AMPK activation by supplement of glucose compromised the sensitivity of HT29 cells to higher dosage of Cet (Fig. 2d-f). However, whether manipulating AMPK activation can overcome the *BRAF* mutation-induced drug resistance might still need further investigated.

AMPK is hypothesized to maintain energy homeostasis by targeting defective mitochondria for autophagy [35] and the regulation of fatty acid metabolism [36]. The activation of AMPK results in the regulation of cell growth, at least in part, through the inhibition of the mTORC1 signaling pathway via the dual phosphorylation of TSC2 and Raptor [28]. Accumulating evidences support the beneficial role of AMPK in gut health mediated through an increase in intestinal absorption, improvement in barrier function, suppression of colorectal carcinogenesis, and reduction of intestinal inflammation and metabolism-related diseases [37]. p-AMPK expression was reported to exhibit a significant prognostic value in a large cohort of CRC patients [38]. Consistent with the results of previous studies, we found that *KRAS* mutation suppressed the activation of AMPK and imparted resistance to anti-EGFR antibody treatment in CRC. The activation of the AMPK pathway by Met or AICAR may overcome the drug resistance induced by *KRAS* mutation. As AMPK activation may exert growth-suppressive effects, the daily intake of Met for decades may lower the incidence of cancer owing to the chronic effects of the AMPK-mediated suppression of mTORC1 and other pro-growth pathways. Today, over 50 different clinical trials are investigating the use of Met in oncology [39]. However, multiple pieces of research also showed that activation of AMPK actually benefits tumor survival and growth [40], and renders drug resistance, including cetuximab [41]. Since the activation of AMPK is also the critical process for cell survival during metabolic stress, it would not be surprising that AMPK activation rewires cancer metabolism to allow cancer cells to survive with inhibition of cetuximab [42]. In our current study, we found the beneficial effect of AMPK agonist in anti-EGFR antibodies only happen in the *KRAS* mutation CRC cells with low activation level of AMPK (Fig. 3e, f). In contrast, continued activation of AMPK in *KRAS* WT CRC cells did not dramatically enhanced the killing effect of anti-EGFR antibodies (Fig. 3e, f). Therefore, the anti-cancer effects of metformin in cancer cells are highly dependent on the AMPK activity, which is correlated to diverse metabolic stress, such as glucose concentration, oxidative stress, and hypoxia level [43, 44]. A further investigation of relationship between AMPK activation level and drug response in different tumor circumstances will be helpful for the usage of AMPK agonists in cancer therapy.

Here we demonstrate that the suppression of AMPK expression by *KRAS* mutation stimulated the mTOR pathway to increase the expression of Mcl-1 at the translational level. AMPK suppresses the mTOR activity directly through the phosphorylation of mTOR at Thr2446 and indirectly through the phosphorylation of TSC2 at Thr1227 and Ser1345, resulting in an increase in the activity of the TSC-complex [28]. It was recently reported that mTOR inhibition specifically sensitizes CRC with *KRAS* or *BRAF* mutations through the suppression of Mcl-1 expression [45]. However, the relationship between the *KRAS* mutation and mTOR pathway activation is still unclear. Here, we found that *KRAS* mutation suppresses the activation of AMPK and consequently increases the activity of mTOR pathway, leading to translation of Mcl-1.

## Conclusions

Overall, we believe that the combination of AMPK activator and anti-EGFR antibodies may mechanistically induce the apoptosis and growth arrest of the subset of CRCs with *KRAS* mutations. Further investigation is warranted to evaluate the clinical outcomes of these molecules in recalcitrant cancers. The current study reveals some interesting findings that may facilitate drug development.

## Supplementary information

**Additional file 1: Fig. S1**. Kras mutated cell showed resistant to anti-EGFR antibodies induced apoptosis. (A) (A) MTS analysis of DLD1 WT (+/−) and Kras mutation (G13D/−) cells treated with Panitumumab (Pan) at the indicated doses for 48 h. (B) Apoptosis in DLD1 WT (+/−) and Kras mutation (G13D/−) cells treated with 10 nM Pan for 48 h was analyzed by nuclear staining with Hoechst 33258. (C) Western blot of p-AMPK, AMPK, and Caspase-3 (C3) in the cells treated as in (B). (D, E) DLD1 WT (+/−) cells stably expressing control, Kras WT, or Kras mutant (G12V) by retrovirus transfection were treated with 10 nM Pan for 48 h. The apoptosis was analyzed by Hochst 33,258 staining (D) and annexin-V staining (E). Each experiment was repeated for 3 times. nd, *p* > 0.05; *, *p* < 0.05; **, *p* < 0.01.

**Additional file 2: Fig. S2.** PUMA is dispensable for AMPK activation induced apoptosis. (A) DLD1 WT cells transfected with control or PUAM siRNA were treated with 5 nM Cet for 48 h. The expression of indicated was analyzed. (B) The apoptosis of DLD1 Kras mutated cells treated as in (A). (C) DLD1 Kras mutated cells transfected with control or PUMA siRNA were treated with 5 nM Cet in combined with 5 μM Met for 48 h. The expression of PUMA and caspase-3 (C3) was analyzed. (D) The apoptosis of DLD1 Kras mutated cells treated as in (C). Each experiment was repeated for 3 times. nd, *p* > 0.05; **, *p* < 0.01.

## Data Availability

The data used to support the findings of this study are available from the corresponding author upon request.

## Abbreviations

CRC: Colorectal cancer
VEGF: Vascular endothelial growth factor
EGFR: Epidermal growth factor receptor
PI3K: Phosphatidylinositol-4,5-bisphosphate 3-kinase
AKT: Protein kinase B
MEK: Mitogen-activated protein kinase
ERK: Extracellular signal-regulated protein kinase
AMPK: AMP-activated protein kinase
STK: Serine/threonine protein kinase
Met: Metformin
Mcl-1: Myeloid cell leukemia 1
mTOR: Mammalian target of rapamycin
shRNA: Small-hairpin RNA
AICAR: 5-aminoimidazole-4-carboxamide ribonucleotide
Bcl-2: B cell lymphoma-2
CHX: Cycloheximide
4EBP1: 4E-binding protein 1
S6K: S6 kinase beta-1
TSC2: Tuberous sclerosis complex 2
siRNA: Small-interfering RNA
TUNEL: Terminal deoxynucleotidyl transferase dUTP nick end labeling
RT-qPCR: Reverse-transcription quantitative polymerase chain reaction
FITC: Fluorescein isothiocyanate
RIPA: Radioimmunoprecipitation assay
FBS: Fetal bovine serum
MET: Metformin
Cet: Cetuximab
Pab: Panitumumab
